# MET overexpression and intratumor heterogeneity in esophageal squamous cell carcinoma

**DOI:** 10.1590/1414-431X2020e10877

**Published:** 2021-05-24

**Authors:** H.S. Abboud, D. Camuzi, D.C. Rapozo, P.V. Fernandes, P. Nicolau-Neto, S. Guaraldi, T.A. Simão, L.F. Ribeiro Pinto, I.M. Gonzaga, S.C. Soares-Lima

**Affiliations:** 1 Programa de Carcinogênese Molecular, Instituto Nacional de Câncer, Coordenação de Pesquisa, Rio de Janeiro, RJ, Brasil; 2 Divisão de Patologia, Instituto Nacional de Câncer, Rio de Janeiro, RJ, Brasil; 3 Departamento de Bioquímica, Instituto de Biologia Roberto Alcântara Gomes, Universidade do Estado do Rio de Janeiro, Rio de Janeiro, RJ, Brasil

**Keywords:** MET, HGF, Intratumor heterogeneity, Esophageal squamous cell carcinoma, Targeted therapy, Biomarker

## Abstract

Esophageal squamous cell carcinoma (ESCC) is among the ten most frequent and deadly cancers, without effective therapies for most patients. More recently, drugs targeting deregulated growth factor signaling receptors have been developed, such as HGF-MET targeted therapy. We assessed *MET* and *HGF* genetic alterations and gene and protein expression profiles in ESCC patients from the Brazilian National Cancer Institute and publicly available datasets, as well as the intratumor heterogeneity of the alterations found. Our analyses showed that *HGF* and *MET* genetic alterations, both copy number and mutations, are not common in ESCC, affecting 5 and 6% of the cases, respectively. *HGF* showed a variable mRNA expression profile between datasets, with no alterations (GSE20347), downregulation (GSE45670), and upregulation in ESCC (our dataset and GSE75241). On the other hand, *MET* was found consistently upregulated in ESCC compared to non-tumor surrounding tissue, with median fold-changes of 5.96 (GSE20347), 3.83 (GSE45670), 6.02 (GSE75241), and 5.0 (our dataset). Among our patients, 84% of the tumors showed at least a two-fold increase in *MET* expression. This observation was corroborated by protein levels, with 55% of cases exhibiting positivity in 100% of the tumor cells. Intratumor heterogeneity was evaluated in at least four tumor biopsies from five patients and two cases showed a consistent increase in *MET* expression (at least two-fold) in all tumor samples. Our data suggested that HGF-MET signaling pathway was likely to be overactivated in ESCC, representing a potential therapeutic target, but eligibility for this therapy should consider intratumor heterogeneity.

## Introduction

Esophageal cancer (EC) is among the most frequent and lethal malignancies in the world, ranking seventh in incidence and sixth in mortality among men (1). This tumor is classified into two histological subtypes: esophageal squamous cell carcinoma (ESCC) and adenocarcinoma. ESCC accounts for approximately 80% of EC cases and has the highest incidence rates in developing countries, such as Brazil.

The high lethality of esophageal cancer is associated with a late diagnosis. Neoadjuvant chemoradiotherapy followed by surgery is the gold-standard treatment for this type of cancer (2), but most patients may not be eligible for this modality due to advanced disease and comorbidities (3). Also, radiotherapy and conventional chemotherapy based on taxane and platinum regimens are ineffective in most cases, commonly showing only a palliative role (4). Therefore, the development of new therapeutic strategies is crucial to improve patients' prognosis.

In recent years, new therapeutic approaches mainly targeting growth factor receptors have been developed for cancer and have shown very positive results for some types of tumors, such as breast and colorectal tumors (5,6). In this context, new strategies are of major interest to improve ESCC treatment and the hepatocyte growth factor (HGF)-MET axis is a promising target. Physiologically, HGF acts as a cytokine and pleiotropic factor that binds to MET, leading to the receptor homodimerization and transphosphorylation of tyrosine residues, present in the intracellular portion of the receptor. As a result, multi-pathway activation takes place and regulates cell proliferation, survival, motility, differentiation, and morphogenesis (7). MET alterations have already been reported in ESCC, including both gene (8) and protein overexpressions (9,10). Moreover, studies have associated MET expression with a poor recurrence-free survival as well as finding it to be an independent predictor of overall survival in ESCC (9,10). Finally, *in vitro* studies have shown that HGF and MET pharmacological inhibition not only reduces the ability of transformed esophageal cells to invade the extracellular matrix but also prompts cell apoptosis and G2/M arrest induced by irradiation (11,12). These findings highlight the potential of HGF/MET-targeted therapies to improve ESCC patients' prognosis.

Although the HGF-MET axis represents a promising target, some barriers for therapy response need to be considered, especially the intratumor heterogeneity, which includes morphological and genetic alterations in different regions of the same tumor mass (13). Cao and colleagues evaluated the mutation profile of patients with ESCC using exome sequencing and comparative array hybridization and described an intratumor heterogeneity rate of 90% (14). However, until this moment no study evaluating HGF-MET dysregulation in ESCC in Occidental populations has been conducted and the intratumor variation of MET expression has never been evaluated.

## Material and Methods

### *In silico* copy number and mutational analysis

*MET* and *HGF* copy number alterations and mutational profiles were obtained from The Cancer Genome Atlas project on esophageal cancer (TCGA, Firehose Legacy). For this, the cBioPortal platform (The cBio Cancer Genomics Portal; http://www.cbioportal.org) was assessed and only ESCC cases (n=96) were selected. The OncoPrint chart with copy number alterations and mutations per sample was generated and downloaded.

### *In silico* microarray analysis

The Gene Expression Omnibus (GEO) database (http://www.ncbi.nlm.nih.gov/geo/) was assessed and three studies including global gene expression data on esophageal squamous cell carcinoma samples and non-tumor surrounding tissues were selected. Raw .CEL files were downloaded from GEO accession numbers: GSE20347 (17 non-tumor and 17 tumor samples) and GSE45670 (8 non-tumor and 28 tumor samples), and GSE75241 (15 non-tumor and 15 tumor samples). The studies used the Affymetrix Human Genome U133 Plus 2.0 Array platform or Affymetrix Human Exon 1.0 ST Array platforms and were processed individually using the software Affymetrix Transcriptome Analysis Console 4.0 (Thermo Fisher Scientific, USA). Summarization was performed by robust multi-array average (RMA) algorithm and gene expression levels by ANOVA (FDR<0.05).

### Human samples

Sixty-six patients with a confirmed diagnosis of ESCC treated at the Brazilian National Cancer Institute (INCA) were included in this study. Fresh-frozen samples (tumor biopsy with at least 70% of tumor cells and adjacent non-tumor biopsy 5 cm from the tumor border) were collected from 37 patients and stored at the National Tumor Bank of INCA (BNT/INCA). Tumor and non-tumor formalin-fixed paraffin-embedded (FFPE) samples were obtained from 29 patients. Fresh-frozen and FFPE samples were from different patients. No patient had undergone chemo- or radiotherapeutic treatment before sample collection.

For the analysis of intratumor heterogeneity, biopsies were collected from five patients submitted to endoscopy at INCA. Two fragments (superficial and profound) were collected from three different regions of the tumor mass: proximal, medial, and distal areas. In addition, two biopsies of adjacent non-tumor tissue were collected 5 cm from the tumor border whenever possible, from proximal and distal esophagus.

The study was approved by INCA's ethics committee and followed the guidelines of the Declaration of Helsinki.

### RNA isolation and RT-qPCR

Total RNA was extracted using the RNeasy Plus mini kit (QIAGEN, Germany), following the manufacturer's instructions. All samples were quantified by spectrophotometry and purity was verified by calculating the absorbance ratio of 260/280 nm and ensuring it was ≥1.7.

A total of 500 ng of RNA was reverse transcribed using SuperScript II^®^ (Invitrogen, USA) following the manufacturer's protocol. The Rotor-Gene Q system (QIAGEN) and QuantiFast SYBR Green PCR Kit (QIAGEN) were used for qPCR, and each reaction was optimized for the specific primers to evaluate the mRNA expression of *MET*, forward: 5′ TTTATTAGTGGTGGGAGCACA 3′, reverse: 5′ TGACATGCCACTGTAAAGTTCC 3′; *HGF*, forward: 5′ TCAGCAAAGACTACCCTAATCAA 3′, reverse: 5′ CAAAAGCCTTGCAAGTGAATGG 3′; and *GAPDH* (as reference gene), forward: 5′ CAACAGCCTCAAGATCATCAGCAA 3′, reverse: 5′ AGTGATGGCATGGACTGTGGTCAT 3′. Each reaction contained QuantiFast SYBR Green Buffer 1X (QIAGEN), 0.5 mM of forward and reverse primers, and sterile deionized water to complete the final volume of 10 µL. After the reaction, the expression of *MET* and *HGF* was normalized with *GAPDH* expression, using the comparative Ct method (15). The number of paired samples varied for each gene evaluation due to the limited amount of available cDNA. Regarding the intratumor heterogeneity analysis, when more than one non-tumor surrounding tissue (NTST) biopsy was available, the mean expression of all non-tumor tissues was used to calculate the expression fold-change in tumors.

### Immunohistochemistry

Freshly cut 4-μm sections of each paraffin block of 22 ESCC samples and 19 NTST were used to perform immunohistochemistry using primary antibody against MET (EP1454Y, Abcam^®^, USA). Antigen retrieval was performed in a water bath while slides were submerged in citrate buffer, pH 6.0, for 40 min at 98°C. The detection was performed following the supplier's recommendations for Novolink™ Max Polymer Detection System (Leica, UK). Samples from lung adenocarcinoma were used as a positive control of MET expression. In the negative control, the primary antibody was replaced with the antibody diluent solution.

Digital images were captured using the Aperio ScanScope CS Slide Scanner (Aperio Technologies, USA) under 20× objective magnification (0.5 μm resolution). An expert pathologist selected the tumor areas using ImageScope software suite (Aperio Technologies). The digital image analysis was performed on whole slide images with Aperio Membrane V9 algorithms (Aperio Technologies). The quantification was done automatically, after algorithm calibration by an experienced observer, and results are reported for MET immunohistochemistry (IHC) as scores from 0 to 3+, and positive tumor cell was defined at the membrane completeness between scores 1 to 3.

### Statistical analysis

All statistical analyses were performed using GraphPad Prism 5 software version 5.02 for Windows (GraphPad Software, USA). Differences were considered statistically significant when P<0.05. Data distribution was assessed by the Kolmogorov-Smirnov normality test. For parametric distributions, ANOVA and paired or unpaired *t*-tests were used. Data with nonparametric distributions were compared using the Mann-Whitney, Wilcoxon, or Kruskal-Wallis test.

## Results

### *HGF* and *MET* alterations through *in silico* analysis

**Figure 1:**
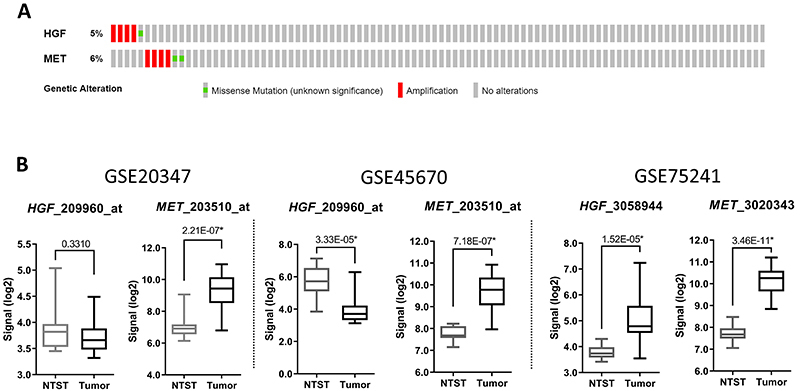
Hepatocyte growth factor (HGF) and MET molecular alterations in publicly available datasets. **A**, OncoPrint chart showing copy number alterations and mutations identified in esophageal squamous cell carcinoma (ESCC) cases from TCGA Firehose Legacy study. Each bar represents a case. **B**, Boxplots showing the expression profile of *HGF* (probe 209960_at or 3058944) and *MET* (probe 203510_at or 3020343) in three publicly available datasets of ESCC samples and non-tumor surrounding tissues (NTST) (GSE20347, GSE45670, and GSE75241). Datasets GSE20347 and GSE45670 used Affymetrix Human Genome U133 Plus 2.0 and dataset GSE75241 used Affymetrix Human Exon 1.0 ST Array. Data are reported as medians and interquartile range. *FDR<0.05 (ANOVA).

The overexpression of growth factor receptors in tumors can be associated with gene amplification and other genetic alterations, so we initially evaluated the copy number and mutational profiles of *MET* and *HGF* in ESCC by assessing TCGA publicly available data (n=96). In total, *HGF* genetic alterations were identified in five patients (5%), with four cases showing gene amplification and one showing a missense mutation of unknown significance (NP_00592.3:p.Arg261Leu); while for *MET*, these numbers were four and two (NP_000236.2:p.Ala327Thr and NP_000236.2:p.Gly672Asp), respectively, totaling six patients (6%) carrying genetic alterations (Figure 1A).

Since genetic alterations were not common in the HGF/MET axis, we evaluated their gene expression profiles in three independent public datasets. All studies showed consistent *MET* overexpression in tumors compared with non-tumor surrounding tissue (fold-change=5.96 in GSE20347; fold-change=3.83 in GSE45670; fold-change=6.02 in GSE75241) (Figure 1B). No significant alterations were observed for *HGF* in GSE20347 dataset, while GSE45670 showed *HGF* downregulation in tumors relative to non-tumor surrounding tissue (fold-change=-3.72), and GSE75241 presented *HGF* overexpressed in ESCC patients (fold-change=2.11) (Figure 1B). Therefore, the next step was to evaluate the expression of these genes in a Brazilian cohort of ESCC patients.

### Patient characteristics

The median age of the patients included in this study was 59.5 years, varying between 40 and 79 years (Table 1). Most of the patients were male (75.8%), ever drinkers (72.7%), and/or ever smokers (78.8%). Tumors affected more frequently the middle third of the esophagus (30.3%), were moderately differentiated (74.2%), and half of patients had tumors diagnosed at early stages (50%). The median overall survival was 9.57 months (0.63 to 165.2 months).

**Table 1:** Social-demographic and clinical-pathologic characteristics of the esophageal squamous cell carcinoma (ESCC) cases included in the study.

Characteristics	ESCC patients, n (%)
Age (median, min-max)	59.5 (40-79)
Gender	
Female	16 (24.2%)
Male	50 (75.8%)
Tobacco smoking	
Never	6 (9.1%)
Ever	52 (78.8%)
Missing	8 (12.2%)
Alcohol drinking	
Never	7 (10.6%)
Ever	48 (72.7%)
Missing	12 (16.7%)
Tumor location in the esophagus	
Upper third	12 (18.2%)
Middle third	20 (30.3%)
Lower third	19 (28.8%)
More than one third	15 (22.7%)
Tumor differentiation	
*In situ*	1 (1.5%)
Well	1 (1.5%)
Moderately	49 (74.2%)
Poorly	12 (18.2%)
Missing	3 (4.5%)
Tumor stage	
0 + I + II	33 (50%)
III + IV	26 (39.4%)
Missing	7 (10.6%)
Survival in months (median, min-max)	9.57 (0.63-165.2)
Techniques performed	
IHC	29 (43.9%)
RT-qPCR	37 (56.1%)

IHC, immunohistochemistry; RT-qPCR: reverse transcription followed by quantitative polymerase chain reaction.

Patient information was available in part of the public datasets used for *in silico* analyses (GSE45670, GSE75241, and TCGA). The median age was 56.5, 60.5, and 57, the most prevalent gender was male (89, 67, and 84%), while the frequency for early diagnosed tumors was 29, 20, and 66% in the GSE45670, GSE75241, and TCGA, respectively.

### *HGF* and *MET* gene expressions in fresh esophageal samples

**Figure 2:**
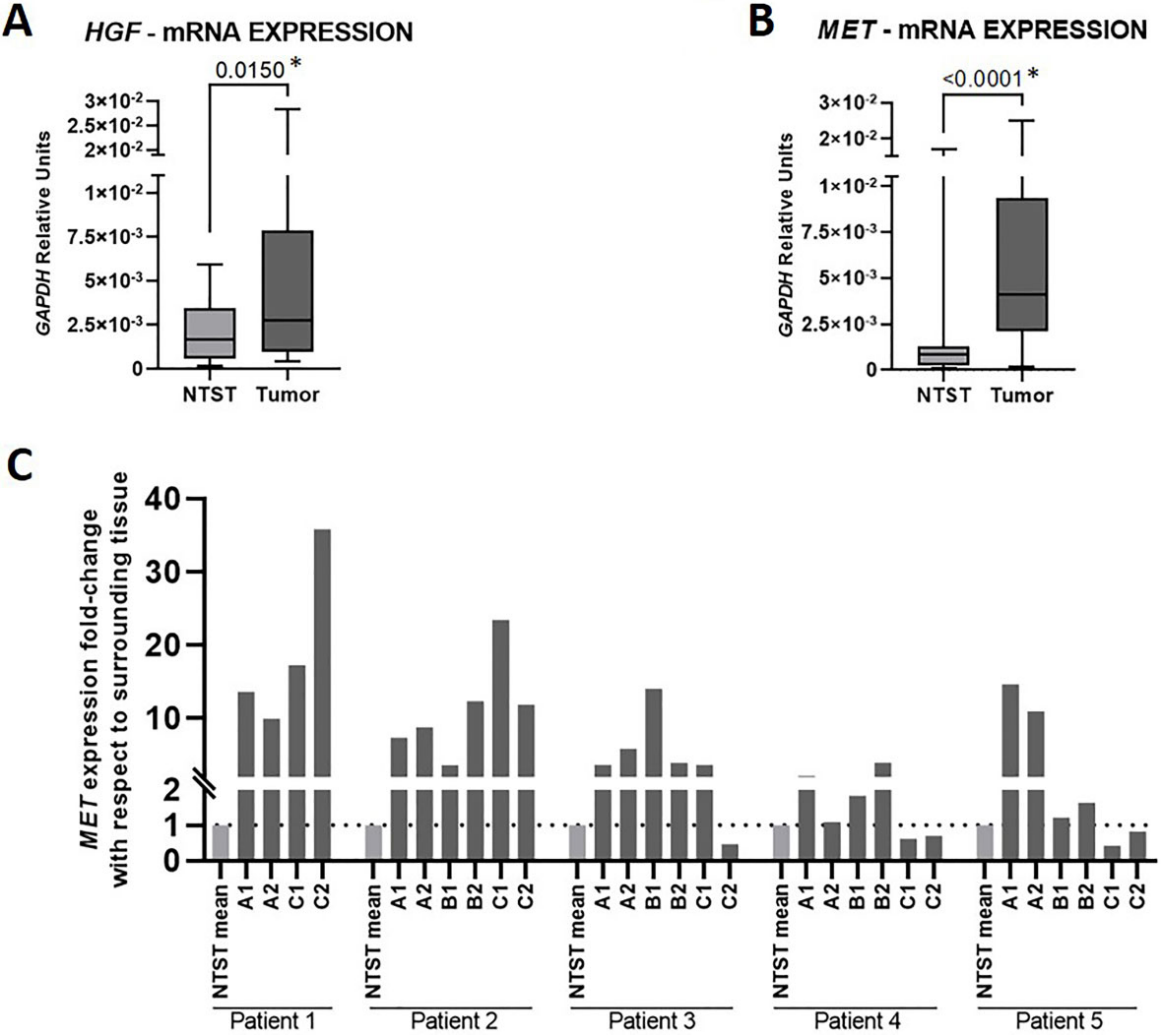
Gene expression of HGF-MET axis in fresh samples from esophageal squamous cell carcinoma (ESCC) patients. **A**, Box-plot showing *HGF* mRNA expression in tumors and non-tumor surrounding tissues (NTST) from 24 ESCC patients. **B**, Box-plot showing *MET* mRNA expression in tumors and NTST from 37 ESCC patients. Data are reported as medians and interquartile range (Wilcoxon test). **C**, Bar graphs showing *MET* mRNA fold-change in each tumor biopsy of five patients with respect to the mean *MET* mRNA expression in NTST.

The analysis of 24 paired samples showed a higher expression of *HGF* in tumors compared with their respective non-tumor surrounding tissue (P=0.015, Figure 2A). The median fold-change between groups was 2.23 (0.29-18.96), with 58% of tumors presenting at least a two-fold increase in *HGF* expression. We also observed *MET* overexpression in ESCC samples in a set of 37 patients (P<0.0001, Figure 2B). The median fold-change for *MET* expression was 5.0 (0.23-27.67), and 83.78% of the cases presented at least a two-fold increase in *MET* expression.

No statistically significant associations between *HGF* (data not shown) and *MET* expression (Supplementary Table 1) and patient socio-demographic and clinical-pathologic data such as age, gender, tumor location, differentiation, and stage were observed. Furthermore, *MET* expression showed no impact on overall survival (data not shown).

Next, we assessed *MET* expression in samples obtained from different tumor regions of five patients, including superficial and profound biopsies. Two out of five patients showed *MET* overexpression (at least two-fold change) in all tumor regions. The other three patients demonstrated a heterogeneous pattern, including some tumor areas with a reduction in *MET* expression compared with NTST (Figure 2C).

### MET protein immunostaining

**Figure 3:**
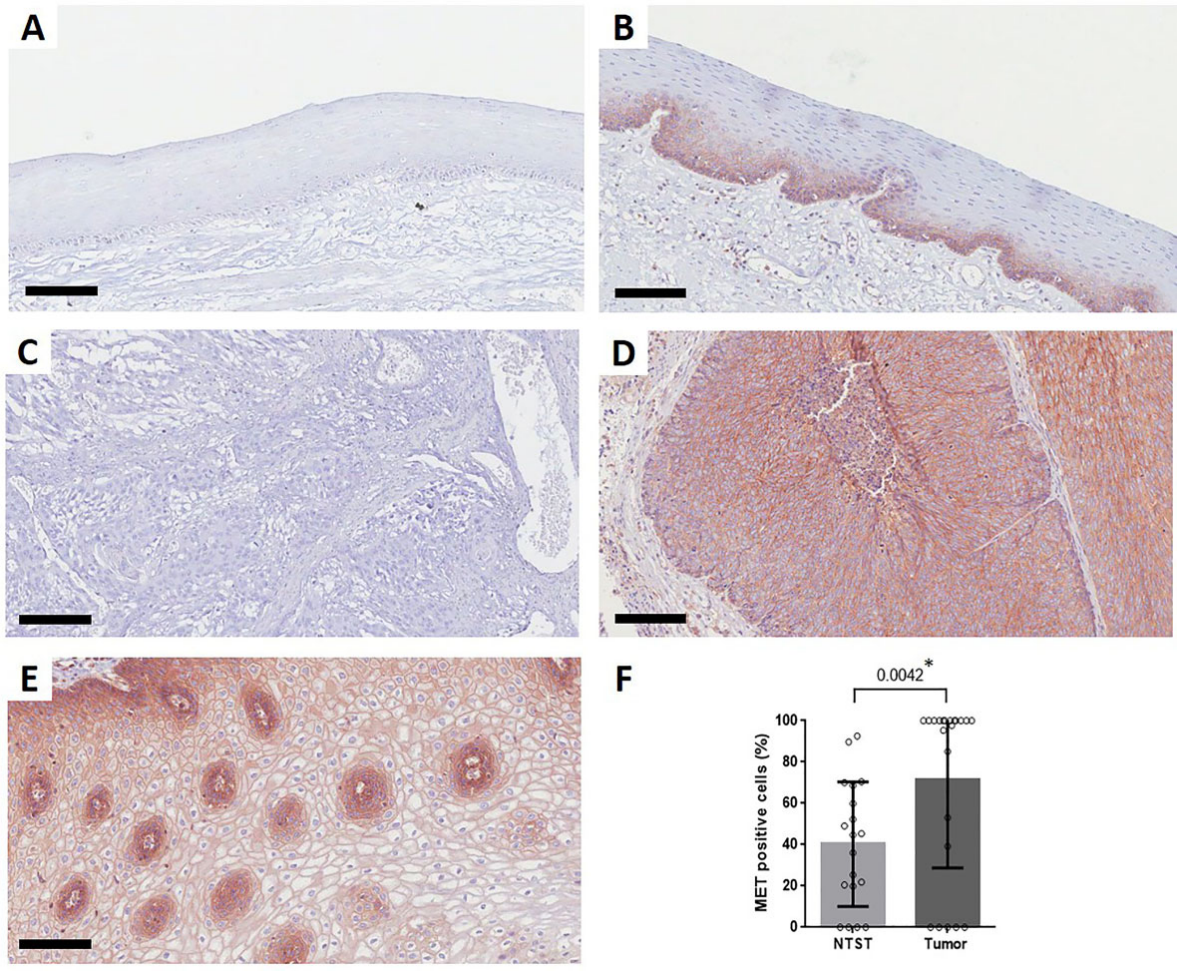
MET immunostaining in esophageal squamous cell carcinoma (ESCC). Representative slides showing a MET-negative (**A**) and a MET-positive (**B**) non-tumor surrounding tissue (NTST) sample, and a MET-negative ESCC sample (**C**). **D** and **E**, Representative slides showing MET-positive ESCC samples that showed membrane staining in 100% of the tumor cells. Scale bars, 100 μm. **F**, Dot-plot showing the percentage of positive cells for MET membrane staining in the NTST (n=22) and ESCC samples (n=25). Data are reported as mean±SD (Mann Whitney test).

MET protein immunostaining was also evaluated in tumor tissues from 22 ESCC patients and NTST from 19 patients, as shown in Figure 3A-E. In all samples, staining was mainly detected in the cell membrane. Among NTST samples, 15 (78.9%) showed MET positive staining, which was mainly observed in the basal and suprabasal layers of the epithelium (Figure 3B). A total of 17 ESCC patients (77.3%) showed positive MET immunostaining in the cell membrane, from which 12 (54.5%) showed positivity in 100% of the tumor cells (Figure 3D-E). The remaining five ESCC samples (22.7%) were negative for MET immunostaining (Figure 3C). Overall, there was a significant increase in MET protein positivity in ESCC (P=0.0042, Figure 3F). No significant associations between the percentage of MET positive cells and socio-demographic and clinical-pathologic data were observed (Supplementary Table 2).

## Discussion

The search for molecular targets is a crucial step to improve and design new therapeutic strategies for cancer types that still rely on conventional chemotherapy as the main therapeutic approach, despite its low efficacy. This is the scenario observed in ESCC, a highly frequent and lethal disease, for which the highest incidence rates are observed in some of the most populous countries in the world, such as China, India, and Brazil (1). Identifying new potential therapeutic targets for ESCC could result in a benefit to a large number of patients, especially from low- and middle-income countries. Currently, the gold-standard treatment for ESCC is neoadjuvant chemoradiotherapy, followed by surgery (2). However, many of the patients are not eligible for surgery due to several factors such as high Performance Status (ECOG-PS), nutritional deficiency, high surgical risk, and advanced disease. Consequently, many patients undergo palliative treatment using chemotherapy and/or radiotherapy (4). Furthermore, few studies on targeted-therapy are being conducted on ESCC, with the focus on epidermal growth factor receptor (EGFR) (16,17). However, recent data have shown that *EGFR* alterations, both mutations and protein overexpression, are not common events in ESCC, which may hamper the success of this therapeutic approach (18,19). In this study, we focused on the evaluation of alterations in the expression of the HGF-MET axis in ESCC, one of the spotlight targets for gastrointestinal (GI) cancer, due to its overexpression in 10 to 70% of different GI tumors (20–22).

The HGF-MET axis orchestrates cell morphology and wound healing in a physiological state, but in cancer, it regulates crucial pathways for tumor growth and metastasis through Ras-MAPK, PI3K, FAK-Src, and STAT3 signaling (7). Alterations in HGF and MET expression are commonly reported in different types of cancers such as non-small cell lung cancer (NSCLC), GI tumors, and hepatocellular cancer (HCC) (23,24). The widespread alterations of the HGF-MET axis boosted the search for their inhibitors in the past decade, which led to FDA approval of two tyrosine kinase inhibitors (TKIs), Crizotinib for non-small cell lung cancer and Cabozantinib for renal cell cancer and medullary thyroid cancer, both capable of inhibiting the altered MET protein activity (25).

*HGF* overexpression is reported in GI tumors and is associated with worse outcomes (22,26). Similar to gastric and colorectal cancer, *HGF* overexpression was observed in almost 60% of the Brazilian ESCC cases from the present study, but it was not associated with patient clinical or pathological features. However, in the two datasets including Chinese patients analyzed here, *HGF* dysregulation was discrepant, either showing no alterations or downregulation in ESCC. These data, together with the low *HGF* amplification frequency reported here, suggest that *HGF* is not commonly upregulated in ESCC.

On the other hand, different studies have reported *MET* overexpression in ESCC, both at gene and protein levels. These observations were corroborated by our findings in an Occidental and in an Oriental population. However, this does not seem to be triggered by copy number gains since *MET* amplification frequency was low in TCGA dataset (6%), as previously described by other authors (ranging from 1-11%) (27–29). However, *MET* was also described to be regulated by other mechanisms, such as the hypomethylation of its promoter in pancreatic cancer, and more than 30 microRNAs regulating *MET* expression, including miR-34a, downregulated in ESCC (30–32). In our Brazilian cohort, *MET* overexpression was highly common, present in 80% of the patients, confirmed by protein immunostaining. MET detection in all tumor cells from more than 50% of the cases agreed with previous reports that showed MET protein overexpression in approximately 45% of ESCC samples (33,34), indicating that MET is a potential therapeutic target for ESCC patients.

However, the failure of MET inhibitors in clinical trials for GI cancer, including MET-positive patients, raised a great deal of distrust about its effectiveness (35). Intratumor heterogeneity might explain, at least in part, these poor results. These trials in GI used a high-score IHC based on ≥50% positive tumor cells, or even ≥25% membrane staining in cancer cells, which still show a predominant heterogeneous pattern in the tumor (12,36,37). It is important to note that patients who present better outcomes in clinical trials with MET inhibitors usually harbor extremely high levels of MET expression (36,38). Also, most cancer treatments are chosen based on a diagnosis from a single biopsy, but recent evidence suggests that tumors are highly heterogeneous not only with respect to microenvironment but also to tumor clones carrying different molecular alterations (39). As a consequence, the development of biomarkers guided by a single biopsy and the use of heterogeneous patterns to classify positive IHC cases may be responsible for the recently reported failures in the implementation of promising therapies in the clinical setting (40). Thus, in the present study, we evaluated the intratumor heterogeneity of *MET* expression through the analysis of biopsies from different tumor regions. When considering the mean expression levels of all tumor biopsies, all patients showed at least a two-fold increased expression in ESCC compared with non-tumor surrounding tissue, but only two out of five cases presented increased expression of *MET* in all individual regions of the tumor. The intratumor patterns of *MET* expression could be an important element to determine a successful response to targeted therapy or to predict possible tumor relapse after treatment and should be better investigated during clinical trials aiming the use of anti-MET drugs in ESCC patients.

Thus, a selection of patients based not only on IHC scores, but also on other evidence of *MET* upregulation has been proposed (24). These arguments together with our data suggest an approach in which ESCC patients with a homogenous overexpression of MET, showing complete IHC positiveness in cancer cells and high *MET* expression in different regions of the tumor mass, would be more likely to respond to anti-MET drugs.

## Conclusions

Our study showed a high frequency of *MET* overexpression in ESCC patients, both in mRNA and protein levels. However, *MET* intratumoral expression patterns were heterogeneous, suggesting this should be considered among eligibility criteria if anti-MET therapy is applied to those cases in addition to IHC analysis. Furthermore, other studies are indispensable to test the potential efficacy of MET-inhibitors in pre-clinical settings with patient-derived xenografts (PDX)-models involving cases with a homogenous MET upregulation. Finally, it is important to highlight that all clinical trials with MET inhibitors on esophageal cancer included mostly adenocarcinoma cases, making ESCC an unexplored field.

## Supplementary Material

Click here to view [pdf].
